# Cortical Network Activity Associated With Different Types of Word Recognition Errors by Older Adults

**DOI:** 10.1162/NOL.a.275

**Published:** 2026-07-28

**Authors:** Mark A. Eckert, Kenneth I. Vaden, Carolyn M. McClaskey, Susan Teubner-Rhodes, Judy R. Dubno

**Affiliations:** Department of Otolaryngology–Head & Neck Surgery, Vagelos College of Physicians and Surgeons, Columbia University Irving Medical Center, New York, NY, USA; Department of Otolaryngology–Head & Neck Surgery, Medical University of South Carolina, Charleston, SC, USA; Department of Psychology, Auburn University, Auburn, AL, USA

**Keywords:** auditory network, cingulo-opercular network, consonant misperception, default mode network, older adults, word recognition error

## Abstract

Word recognition scores are useful general metrics of speech understanding but provide limited insight about why listeners exhibit difficulties with word recognition tasks. For example, older adults can have the same word recognition score but make different types of errors. Different types of misperceptions for audible speech, such as misunderstanding the final consonant or a whole word, suggest that word recognition errors reflect distinct neural failures in speech processing. Patterns of activity in central auditory and attention-related cortical networks were hypothesized to predict different types of word recognition errors. Trial-level error analyses were performed with data from two neuroimaging experiments where older adults performed word recognition in noise tasks (Data Set 1: *N* = 45, mean age = 64.71 years; Data Set 2: *N* = 40, mean age = 68.97 years). Group-level independent component analysis of fMRI data collected during these tasks identified distinct patterns of activity in auditory and cingulo-opercular networks that were associated with initial or final consonant substitution errors. In addition, complete word omission errors occurred with relatively low activity in the auditory network and elevated activity in cingulo-opercular and/or default mode networks. These findings suggest that some errors occur with more cingulo-opercular-related performance monitoring, whereas other errors occur with insufficient signal in auditory cortex and/or task disengagement that has been associated with default-mode network function. Thus, patterns of cortical activity reflect the type of word recognition error and may inform reason(s) for those errors that may not be evident in an overall word recognition score.

## INTRODUCTION

The ability to accurately understand speech in the presence of competing sounds such as background noise is a fundamental aspect of spoken communication that declines with age (Dubno et al., 1984). Reasons for the speech recognition in noise difficulties of older adults may include auditory system declines (Başkent, 2006; McClaskey, 2024; Ouda et al., 2015) and/or declines in higher cortical functions that support performance during challenging tasks (Alain et al., 2018; Dryden et al., 2017; Eckert et al., 2016; Moore et al., 2014; Wong et al., 2010). Poor speech recognition is characterized by different types of perceptual errors, such as misidentifying a word or failing to understand part of a word or a phoneme (e.g., hearing “pat” when “bat” was spoken). Atypical trial-level activity across neural networks during speech sound processing may explain why listeners make different types of word recognition errors. Although there is a growing understanding of the neural networks that support speech understanding in noise (Peelle, 2018), the neural activity that occurs with and may contribute to specific error types is less well understood. The current study was designed to address a gap in our understanding about the role of cortical networks that support speech understanding when older adults make different types of word recognition errors.

There is a classic and rich literature cataloguing different types of speech recognition errors in various listening conditions, which demonstrates that final consonants are more likely to be misperceived than other speech sounds (Dubno et al., 1982; Miller & Nicely, 1955; Oyer & Doudna, 1959). The type of speech recognition error also appears to depend on the listening condition. For example, listeners were more likely to omit a word when repeating sentences presented in a steady-state noise compared to fluctuating noise (Smith & Fogerty, 2017). This whole word omission error was also more likely among older than younger adults who had difficulty recognizing speech in noise (Smith & Fogerty, 2021). These errors are thought to occur with limited access to speech cues because of degradation by the presence of noise, reduced speech audibility due to hearing loss, and declines in processing abilities throughout the auditory system (Baskent & Shannon, 2003; Bidelman et al., 2014; Harris et al., 2021; Humes & Dubno, 2010), as well as age-related changes in cognitive functions that are necessary for maintaining a representation of speech stimuli (Dryden et al., 2017; Smith & Fogerty, 2021).

Neuroimaging studies on speech recognition in noise have demonstrated a consistent pattern of elevated activity in attention-related cortex that supports speech recognition in challenging listening conditions (Alain et al., 2018; Eckert et al., 2016). For example, younger and older adults demonstrated elevated activity across cingulate, inferior frontal gyrus, and anterior insula regions in word recognition in noise tasks (Eckert et al., 2022; Erb & Obleser, 2013; Vaden et al., 2013). This cingulo-opercular pattern of activity is consistent with a performance monitoring response that has been observed across behavioral tasks (Botvinick et al., 2004), even when listeners do not receive feedback about when they made an error (Vaden et al., 2013, 2022). However, the degree to which specific types of word recognition errors contribute to a cingulo-opercular response is unclear. Cingulo-opercular activity may be increased when listeners know that they have made an error, such as when listeners omit a response, but perhaps to a lesser extent when they misperceive the consonant or report hearing an additional phoneme because of an increase in background noise occurring at the end of a word (e.g., “bats” when “bat” was presented). These single-speech-sound misperceptions might also be expected to occur with relatively more activity in auditory cortex, which exhibits increased activity with increased speech intelligibility (Davis & Johnsrude, 2003; Eckert et al., 2008; Erb et al., 2013; Scott et al., 2000; Venezia et al., 2019). That is, elevated cingulo-opercular function and/or lower auditory cortex function during speech processing may reflect the extent of perceptual uncertainty across different types of word recognition error.

Because attention is important for speech recognition in noise (Peelle, 2018), the type of speech recognition errors may also reflect listeners’ ability to sustain attention during speech recognition tasks. That is, listeners with drifting attention or a lack of task focus, which appears more likely when hearing speech in noise compared to quiet listening conditions (Steindorf et al., 2023), would be expected to have difficulty hearing every phoneme of a word. Such task disengagement is represented by patterns of activity that can be measured by EEG or fMRI (Eqlimi et al., 2020; Poerio et al., 2017). This pattern of activity includes increased activity in a default mode network (DMN) composed of brain regions that include ventromedial prefrontal, precuneus, and bilateral parietal cortex (Bowman et al., 2017; Christoff et al., 2009; Kucyi et al., 2016; Mittner et al., 2014; Mo et al., 2013; Poerio et al., 2017). Increased activity in the DMN generally occurs during internally generated thoughts and a shift in attention away from a task or from engagement with the environment (Menon, 2023; Raichle, 2015). Elevated DMN activity may also reflect semantic- or conceptual-level processing of connected speech involving memory retrieval (Fernandino & Binder, 2024; Liu et al., 2022; Wirth et al., 2011). However, it is not clear that DMN activity supports word recognition in noise because DMN activity appears to be relatively suppressed during more difficult compared to easier listening conditions (Erb & Obleser, 2013). That is, elevated DMN function may contribute to word recognition errors and occur with word omissions when listeners are disengaged from the listening task or may reflect semantic processing during word recognition.

The current study was designed to test the overarching hypothesis that different types of word recognition errors occur with different patterns of brain activity. Given lower auditory network activity and higher cingulo-opercular network activity when speech intelligibility is low (Alain et al., 2018; Eckert et al., 2016; Peelle, Johnsrude, & Davis, 2010), we hypothesized that whole word omission and vowel errors would be more likely to occur with relatively low auditory network activity and high cingulo-opercular network activity because of an impoverished central auditory signal that limits perception and increases task demand. We also hypothesized that these errors could occur with more DMN activity, particularly in listeners with lower cognitive function, such as slower processing speed and poorer sustained attention, which may contribute to task disengagement. In contrast, consonant substitution errors (e.g., “pool” when “cool” was presented) and additive phoneme errors (e.g., “posed” when “pose” was presented) were hypothesized to occur with relatively lower cingulo-opercular network activity. The following results, which were replicated across two different samples of middle-aged and older adults, demonstrate that different word recognition errors occur with distinct patterns of cortical activity that can inform why people experience speech recognition in noise difficulties and why they make specific errors.

## METHODS

### Participants

This study included data from two different fMRI experiments that examined the neurobiology of word recognition in noise among middle-aged and older adults. Data Set 1 participants were recruited to study word recognition in noise when visual cues were provided so that participants could anticipate relatively easier or harder signal-to-noise ratio (SNR) conditions (*N* = 40, 26 women, *x̄* = 68.97 ± 6.54 years of age, 17.24 ± 3.19 years of education). Data Set 2 participants were recruited to study voicing and pitch influences on word recognition in noise (*N* = 45, 32 women, *x̄* = 64.71 ± 7.31 years of age, 16.40 ± 4.53 years of education). Participants in both studies were native English speakers who reported no history of neurological or psychiatric events and had no evidence of conductive hearing loss. Participants were also excluded if they had claustrophobia or ferrous metal in the body that are contraindicative for MRI scanning. Informed consent was obtained, and the Medical University of South Carolina Institutional Review Board approved the research that was conducted in accordance with the Declaration of Helsinki.

**Figure 1.**
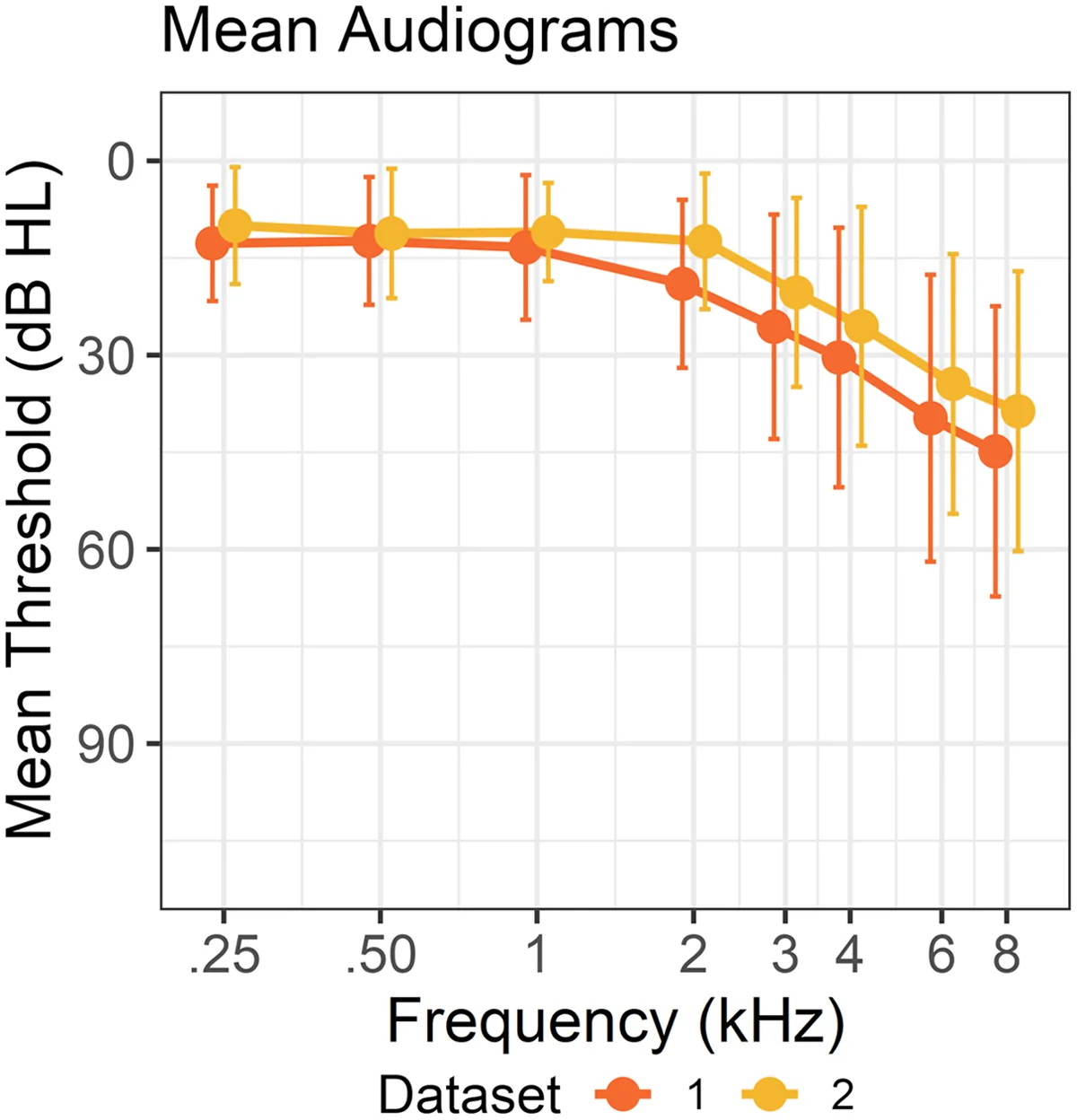
Mean audiogram from each data set with standard deviations. Thresholds from the ear with the best pure-tone average were used for these plots. Error bars indicate standard deviation.

Audiometric testing was performed to limit the influence of audibility differences in the word recognition in noise tasks. The hearing-related criteria for the current study were pure-tone thresholds <40 dB HL from 0.25 to 2 kHz, <55 dB HL at 3.0 kHz, <70 dB HL at 4.0 kHz, and <90 dB HL at 8.0 kHz across both studies. These criteria were chosen such that participants from this age range would be in the upper 50% for hearing sensitivity based on the median thresholds from middle-aged to older adults in a longitudinal cohort study of age-related hearing loss (Vaden et al., 2017). Figure 1 presents the summary audiogram for each data set. A better ear pure-tone average (PTA; 0.25–8.0 kHz) was also used to determine the extent to which hearing sensitivity impacted the word recognition results. The mean absolute PTA difference between ears was 3.14 and 3.28 dB HL in Data Sets 1 and 2, respectively.

### Cognitive Assessment

Cognitive constructs that could impact speech recognition in noise were also collected in this study. These constructs included (1) cognitive processing speed, where faster processing speed has been consistently related to better speech recognition in noise (Dryden et al., 2017); (2) set-shifting, where better set-shifting has predicted better speech recognition in noise (Eckert et al., 2024) and elevated cingulo-opercular activity (Teubner-Rhodes et al., 2017); (3) inhibitory control, which is likely critical for selective attention and tracking semantic context in connected speech tasks (Dey & Sommers, 2015; Peelle, 2018; Shinn-Cunningham & Best, 2008) but could also explain individual differences in word recognition and has been related to increased activity in attention-related cortex (Freitas et al., 2023), particularly among older adults (Milham et al., 2002); (4) sustained attention because the ability to maintain attentional focus can be important in the context of long-duration listening tasks (Zhao et al., 2019); and (5) vocabulary knowledge that could impact the ability to retrieve words and consider competing alternative responses (Carroll et al., 2016).

#### Processing speed

Cognitive processing speed was measured using the Connections Cognitive Processing Speed Test (Salthouse et al., 2000), in which participants are instructed to manually connect lines between sequences of letters or numbers on a page (Simple processing speed; e.g., 1, 2, 3 …) or alternate between letters and numbers (Complex processing speed; e.g., 1, A, 2, B, 3, C …) as quickly as possible in 20 s. Thus, the task assesses motor and perceptual processing speed, with additional working memory and response inhibition demands assessed during the Complex test. Participants completed two pen-and-paper versions each of the Simple letters, Simple numbers, Complex letters–numbers, and Complex numbers–letters tasks. Each set of Simple and Complex scores was averaged to obtain measures of processing speed without or with inhibitory control and working memory demands, respectively, where a higher number of connections indicates faster processing speed.

#### Set-shifting

Set-shifting was measured using the Wisconsin Card Sorting Task-64 (Greve, 2001; Kongs et al., 2000), in which participants had to determine whether to sort cards by color, shape, or number of shapes displayed on the cards and then identify a new sorting strategy when the rule changed after the participant demonstrated understanding of the sorting rule. Thus, the task assesses working memory, attention, problem solving, and performance monitoring. The overall measure of correct number of card-sorting trials was used in the current study, where a higher value reflects better set-shifting.

#### Inhibitory control

The Stroop Neuropsychological Screening Test was used to assess the ability to inhibit cognitive interference (Trenerry et al., 1989). Participants were asked to name the color of the font that was used to present a color word (e.g., the word “red” in blue font). Participants had to suppress a prepotent response to read the word and instead name the font color. The total number of correct trials was used as the measure of inhibitory control.

#### Sustained attention

The Connor’s Continuous Performance task (Conners, 2000) was used to measure sustained attention and, more specifically, inattention and impulsivity, as defined by errors of omission and commission, respectively. Participants were shown letters on a computer monitor, and they pressed the space bar on a keyboard following the presentation of each letter, except when the letter “X” appeared. Errors of omission were observed when participants failed to button-press for non-X letters. Errors of commission were observed when participants button-pressed after an “X” was presented. Higher values for errors of omission and commission indicate poorer sustained attention.

#### Vocabulary knowledge

The Wechsler Test of Vocabulary Knowledge was administered to test how well participants can define and understand words (Wechsler, 1999). Participants were asked to define words by identifying the main category of the word, its features or functions, or by giving a specific example of the word. Participants earned between 0 and 2 points on each item based on the quality of their response, for up to 80 points in total across 40 words. Higher scores reflect better vocabulary knowledge.

### Word Recognition fMRI Tasks

#### Common methods across tasks

Participants were administered consonant–vowel–consonant word recognition in noise tasks, where the words and speech-shaped background noise were presented binaurally through Sensimetrics piezoelectric insert earphones using E-Prime (Psychology Software Tools). On each trial, participants were instructed to listen to and then repeat the word they heard when visually presented with a red center crosshair or to say “nope” if they could not understand the presented word and generate a response. This design choice was made to ensure that there was a vocal response on every task trial of these functional imaging experiments so that analysis of the brain activity data was not confounded by the absence of motor-related activity. This approach also ensured that participants were engaged in the tasks rather than resting quietly in the scanner. Participants did not receive feedback about their word recognition accuracy during the tasks.

Both fMRI experiments were administered using a sparse sampling image acquisition design that allowed for the presentation of auditory stimuli and collection of participant responses in periods of silence between the acquisition of image volumes (Perrachione & Ghosh, 2013). This approach prevents scanner noise from interfering with speech recognition, allows participants time to stabilize their head before collection of the next image volume, and can efficiently collect the relatively slow BOLD contrast measure of brain activity in response to the word presentations. All auditory stimuli were low-pass filtered at 3.15 kHz so that individual differences in high-frequency hearing sensitivity would have limited impact on differences in speech recognition. In addition, participants practiced each experiment prior to MRI scanning so that they understood the timing of presentations and what they needed to do during the task. Both experiments included non-task rest trials at the beginning, middle, and end of the experiment to balance the rest periods over time and to allow time for participants to acclimate to the scanner and rest between blocks. Instructions in both experiments were to listen for a word presented in background noise and, when a center crosshair turned red, repeat the word they heard or say “nope” if they could not understand the word.

Features specific to each of the word recognition experiments are described next. This includes an open-set word recognition task design in Data Set 1 compared to the relatively more closed-set /b/ and /p/ initial consonant task design in Data Set 2. While there were differences between the data sets, including with Data Set 1 words having higher word frequency, *t*(122) = 2.405, *p* = 0.018, and phonological neighborhood density, *t*(122) = 4.122, *p* < 0.001, than Data Set 2, the current study focused on common error effects across experiments, including in the context of statistical testing described later.

#### Data Set 1: Cued listening in noise

Participants were administered a word recognition task that was designed to examine cueing effects on brain activity. The 68 words presented in Supplementary Table S1 (Supporting Information can be found at https://doi.org/10.1162/NOL.a.275) were selected from the Northwestern University Auditory Test No.6 (NU-6) (Tillman & Carhart, 1966) and presented individually on each trial at 84 or 92 dB SPL, mixed with speech-shaped noise at 82 dB SPL (+2 and +10 dB SNR, 136 trials). The noise was shaped to the spectra of the average frequency spectrum of the concatenated NU-6 word stimuli using functions from the seewave (Version 2.1.5; Sueur et al., 2008) and tuneR (Version 1.3.3; Ligges et al., 2013) libraries for R. A visual cue was presented to inform participants about the relative listening difficulty of each word recognition trial based on SNR. For half of the trials, an upward pointing triangle was presented during the easier +10 dB SNR trials and a downward pointing triangle was presented during the more difficult +2 dB SNR trials. A square was presented on the remaining half of trials as a neutral or uninformative cue about listening difficulty. The visual symbols were presented at the onset of each trial, 3.1 s before the word onset, to serve as an anticipatory cue. Words were presented twice across different trial blocks in the same SNR condition so that each word was presented with both an informative and an uninformative visual cue during the experiment.

#### Data Set 2: Voicing and pitch conditions in noise

Participants were administered a word recognition task that was designed to examine voicing and pitch effects on brain activity. The 52 words presented in Supplementary Table S2 are labial plosive initial consonants (/b/ or /p/), consisting of minimal pairs that differed in voicing and were presented in two pitch conditions (100 trials). The words were recorded by a male speaker, then pitch-shifted by ±3 semitones to create a high-pitch and a low-pitch condition using Praat (Version 6.2). These voicing and pitch manipulations were used to vary word recognition difficulty and determine if participants had more difficulty recognizing words with a shorter voice onset time and higher pitch. All words were presented at 88 dB SPL with a speech-shaped background noise at 73 dB SPL (+15 dB SNR). As in Data Set 1, the background noise was shaped to match the average frequency spectrum of the concatenated word stimuli.

### Word Recognition Error Item Analyses

Word recognition responses were transcribed in real time by two raters during the fMRI experiments. A digital recording of the participant responses was also collected during each scanning session using an MRI-compatible microphone (Resonance Technology) attached to the scanner head coil. These recordings were used to establish rater consensus when there was inconsistent transcription of participant responses (9.49 % in Data Set 1, 6.00 % in Data Set 2).

The error word transcriptions and the presented word were analyzed in R (Version 4.2.1), which was used to identify errors that occurred on the initial consonant, final consonant, medial vowel, the inclusion of extra or additive phonemes, complete word substitution, and omissions. The Carnegie Mellon University (CMU) Pronouncing Dictionary was used to convert the word transcriptions to machine-readable phonemes (Weide, 1998). Comparison of phonemes between the presented word and the error response determined which type of error occurred, as defined next.

#### Initial consonant errors

Initial consonant errors were defined as substitution errors in which the initial consonant of each word was different from the target, but all other phonemes matched. Because the vowel and final consonant matched the target, these errors occurred when the listener responded with a word that rhymed with the target word. For example, the response “pun” was recorded as an initial consonant error when “bun” was presented. Initial consonant omissions, defined as errors in which the initial consonant was excluded from the response but all other phonemes were accurate, were excluded from the initial consonant error labeling because of their infrequency (Data Set 1: *N* = 5 trials across 40 participants; Data Set 2: *N* = 1 trial across 45 participants).

#### Final consonant errors

Final consonant errors were defined as substitution errors where the final consonant was incorrect, but all other phonemes matched between the target and response. For example, the response “path” was recorded as a final consonant error when the word “pass” was presented. Final consonant omissions, defined as errors in which the final consonant was omitted but all other phonemes were correct, were excluded from the final consonant error labeling because of their infrequency (Data Set 1: *N* = 14 trials across 40 participants; Data Set 2: *N* = 13 trials across 45 participants).

#### Vowel errors

Vowel errors were defined as substitution errors in which the vowel sound differed between the target and the response, but the initial and final consonants matched. For example, the response “bowl” was recorded as a vowel error when the word “bull” was presented. These pure vowel errors were relatively infrequent (Data Set 1: *N* = 11 trials across 40 participants; Data Set 2: *N* = 41 trials across 45 participants). Compound vowel errors were also recorded when there was also an error for the initial or final consonant. The pure vowel errors exhibited similar network activity effects to the compound vowel errors and are not shown.

#### Additive errors

Additive errors were recorded when participants added a phoneme to the beginning or ending of a word. These errors were calculated by determining if the target word was contained within the participant’s response. For example, the response “posed” was recorded as an additive error when the word “pose” was presented. As shown later, these were also relatively infrequent errors and motivated grouping initial and final consonant additive errors into the same error category.

#### Whole word substitution errors

Whole word substitution errors were recorded when none of the CMU pronunciation phonemes in a participant’s response overlapped with the phonemes in the presented word. For example, the word “maw” was recorded as a substitution error when the word “bop” was presented.

#### Whole word omission errors

Again, participants were instructed to report “nope” when they did not understand a word and could not make an informed guess about what they heard. These nope responses were coded as whole word omission errors. Trials during which participants failed to produce a response were excluded to ensure that the analyses were limited to trials when participants were sufficiently alert to respond in the scanner and to ensure that a speech-motor response was produced on every trial.

### Imaging Procedures

#### Image acquisition

Functional and structural scans were collected using a 3T Siemens Prisma system with a 32-channel head coil. Functional imaging (fMRI) data were collected using a single-shot echoplanar imaging sequence to obtain BOLD images with the following parameters: Data Set 1, TR = 8,600 ms, TE = 35 ms, TA = 1,535 ms, 90° flip angle, 36 slices, 3.0 mm thickness, multiband acceleration factor = 2; Data Set 2, TR = 8,600 ms, TE = 40 ms, TA = 1,630 ms, 46° flip angle, 56 slices, 2.5 mm thickness, multiband acceleration factor = 4. T1-weighted MP2RAGE structural images were collected following the completion of the fMRI tasks with the following parameters (Marques et al., 2010): TR = 5,000 ms, TE = 2.98 ms, TI = 700/2,500 ms, flip angle of 4°/5°, 176 slices in a 256 × 256 data matrix with 1 mm slice thickness and no slice gap. fMRI images were co-registered to MP2RAGE structural images to facilitate group statistical analyses in the same anatomical space.

#### Image preprocessing

A sample-specific anatomical template was created for each data set from the T1-weighted MP2RAGE images using Advanced Normalization Tools (ANTs Version 1.9; Avants et al., 2009). Each template served as the anatomical space for performing group-level analyses using the functional imaging data. The functional imaging data were processed using SPM12 (www.fil.ion.ucl.ac.uk/spm) default settings to realign, unwarp, and then co-register each participant’s images to their T1-weighted anatomical image. The ANTs normalization parameters that were obtained for each participant when creating the sample-specific template were used to spatially normalize the functional images, as in our previous studies (Vaden et al., 2013, 2022). SPM12 was used to spatially smooth the normalized functional images using a Gaussian kernel (8 and 7 mm full-width half-maximum because of the 3 and 2.5 mm isometric voxel sizes of each data set, respectively).

To measure the auditory, default mode, and cingulo-opercular networks that were a focus of this study, Independent Component Analysis (ICA) decomposition was applied to the fMRI data using the Group ICA of fMRI Toolbox (GIFT 4.0b) with default parameters, back-reconstruction, and the ICASSO stability function (Erhardt et al., 2011). This analysis generated a set of independent components in the form of a time series of brain activity for each participant that characterizes common temporal and spatial patterns of activity across participants. This approach significantly reduces the dimensionality of the data and thus excludes noise in the data (Calhoun et al., 2001). The canonical auditory, default mode, and cingulo-opercular patterns of activity examined in this study have been consistently observed across task-based and resting-state fMRI studies using independent component or similar covariance approaches (Calhoun & Adalı, 2012; Iraji et al., 2023; Menon & D’Esposito, 2022). Visual inspection of the independent component results was used to identify these patterns of activity, which are shown later to demonstrate consistency with the same patterns of activity within this study and for comparison with the extant literature. The time series from these independent components were collected for each participant and used in trial-level analyses described later to examine when variation in network activity occurred with each different word recognition error type. That is, the time series reflected the fluctuation in the magnitude of activity over time for each network or independent component.

To further limit the impact of noise on the network-level time series, trial-level head rotation and translation measures were calculated from the SPM realignment parameters that were generated during head motion correction (Kuchinsky et al., 2012; Wilke, 2012) and then residualized out of the participant-level time series. These motion-adjusted time series were then high-pass filtered (128 s) using FIACH package (Version 0.1.2; Tierney et al., 2016) in R (R Core Team, 2010) to avoid the potential impact of low-frequency drift on the statistical results. All statistical analyses excluded trials where the functional volume had an average intensity value >2.25 *SD* from the average intensity of each participant’s set of image volumes, as reported in Eckert et al. (2022).

We next examined the degree of spatial overlap between GIFT networks from each data set. Using the GIFT *t*-test function, a one-sample *t* test was conducted across participants to functionally define network spaces for each data set. The resulting images were then thresholded using a family-wise error corrected (*p*_FWE_ < 0.05) to create a binary image of each network, which was then warped into MNI coordinate space using the ANTs antsRegistrationSyNQuick and antsApplyTransforms functions to spatially compare the networks in the same coordinate space. Finally, the MNI transformed and thresholded binary network masks were correlated within MATLAB (MathWorks) using the corrcoef function.

### Statistics

R (Version 4.2.1) was used for the following statistical analyses.

Descriptive statistics and data set comparisons were first performed to characterize demographic, cognitive, and error type similarities and differences between the two data sets. Multiple-comparison corrected correlation tests were performed to determine the extent to which the relative occurrences of each error type were associated with demographic and cognitive measures. These analyses provided evidence about the impact of cognitive abilities on the type of errors that listeners are likely to make compared to other errors.

#### Network activity hypothesis testing

Generalized linear mixed models (GLMMs)-based regression tests were used for hypothesis testing to examine network activity and error type associations. The first set of analyses focused on auditory and cingulo-opercular network time series. Trial-level neural activity, or BOLD contrast time series (GIFT-ICA components), was included as the independent variable, and each error type was included as the dependent variable, with a subject random effects term. Separate analyses were performed for each error type (initial consonant, final consonant, voicing, additive, whole word substitution, and whole word omission) and for each network (auditory, cingulo-opercular, DMN). Comparisons were performed for each error type relative to correct trials to test the hypotheses, for example, that omission errors but not initial consonant errors occur with more cingulo-opercular activity than correct trials. GLMM tests were then used to compare each error type to all other error types, which determined the extent to which each error type exhibited a unique pattern of auditory and cingulo-opercular activity compared to all other error trials.

The same correct and error trial analysis approaches were used to examine DMN activity. Relatively more anterior or more posterior patterns of DMN activity, as consistent with the literature (Kim & Lee, 2011; Liu et al., 2022; Taylor et al., 2023), were identified in both data sets. Before integrating the DMN variables with the auditory and cingulo-opercular network data, these anterior and posterior DMN time series were included as independent variables in the same GLMM to examine the specificity of their effects on word recognition errors. These analyses were also performed separately from the auditory and cingulo-opercular analyses to test the competing hypotheses that anterior and posterior DMN activity is elevated during whole word omission trials rather than elevated during accurate word recognition, which would be expected if additional language operations were performed on the stimuli (e.g., semantic processing). For effects that were observed in both data sets, follow-up analyses were then performed to determine the extent to which DMN effects remained significant when auditory and cingulo-opercular time series were included in the analyses.

#### Multiple comparison correction

To control for false positive results, a two-step procedure was used to determine statistical significance. First, Bonferroni correction was used to set the statistical threshold for an initial screening of the GLMM time series results (*p* = 0.05 / 6 error types = *p* < 0.0083). Second, this threshold was required to be met in both data sets for a given comparison to define a significant association between an error type and network activity.

## RESULTS

### Data Set Comparisons

Table 1 shows that participants in the two data sets did not exhibit significant differences in years of education, sex, better ear PTA, cognitive processing speed, or vocabulary knowledge. The participants in Data Set 1 were 4.26 years older than those in Data Set 2. Table 1 also shows that Data Set 1 participants had lower set-shifting but better inhibitory control compared to Data Set 2 participants.

**Table 1.** Demographic and cognitive comparisons between data sets.

	Data Set 1		Data Set 2		Difference estimates	
	*n*	%	*n*	%	*χ* ^2^	*p*
Sex					0.14	0.711
Female	26	65	32	71		
Male	14	35	13	29		
	***M***	***SD***	***M***	***SD***	***t***	***p***
Age (years)	**68.97**	6.54	**64.71**	**7.31**	**−2.81**	**0.006**
Education (years)	17.24	3.19	16.40	4.53	−0.86	0.394
Better Ear PTA (db HL)	24.77	12.78	20.46	10.97	−1.67	0.098
Simple	23.51	5.49	24.16	6.90	0.48	0.635
Complex	13.28	3.73	13.92	4.55	0.71	0.483
Inhibitory control	**100.19**	**13.88**	**83.54**	**23.96**	**−3.70**	**<0.001**
Set shifting	45.08	10.88	49.19	6.05	2.11	0.039
Vocabulary	65.43	7.64	68.67	8.08	1.88	0.064

*Note*. Better ear pure-tone average (db HL, average of thresholds from 0.25 to 8 kHz). **Bold** text indicates factors that were significant predictors in both data sets after Bonferroni correction for multiple comparisons (*p* = 0.05/8 comparisons).

### Word Recognition Error Patterns

**Figure 2.**
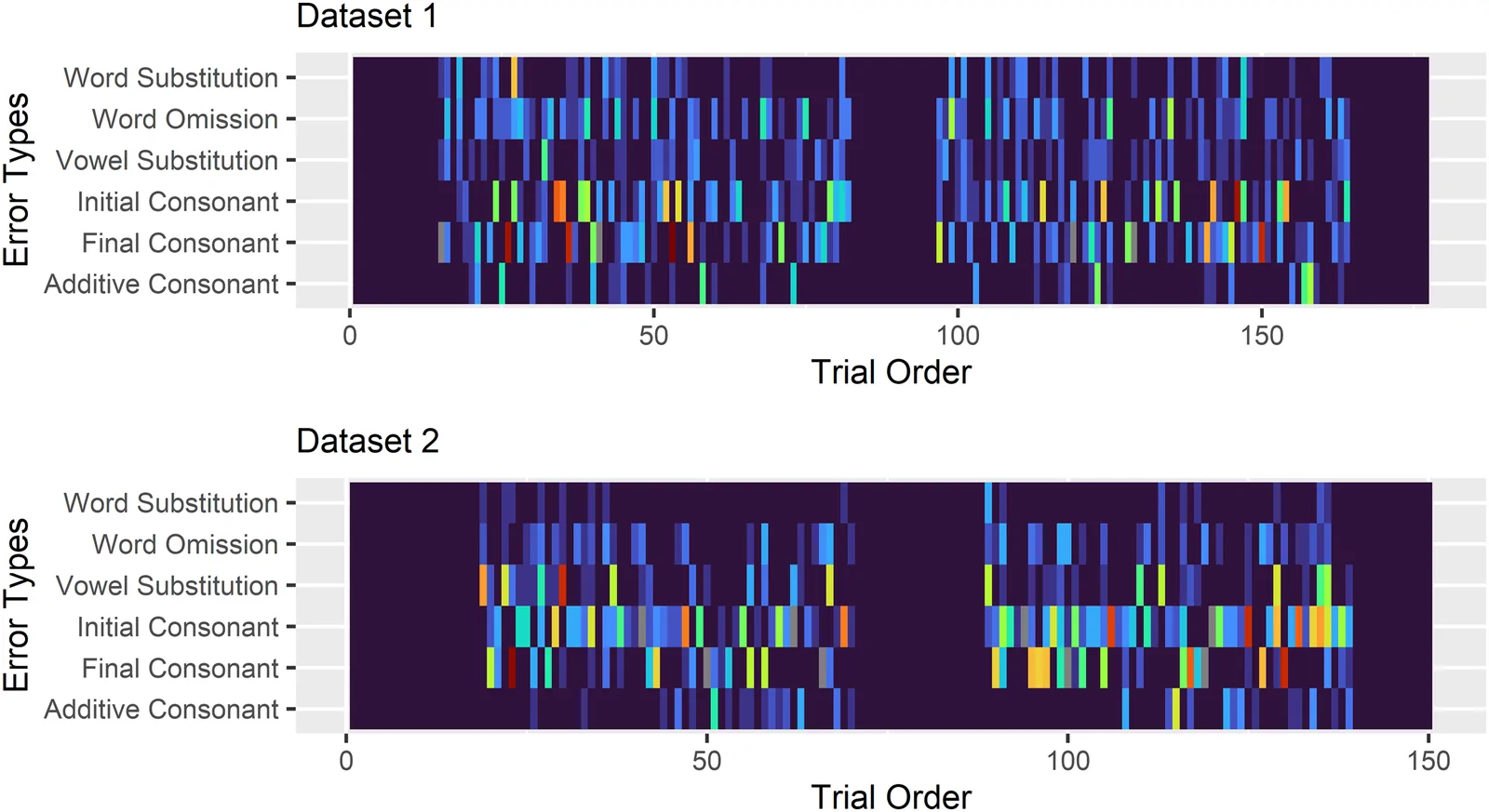
Word recognition error types across each word recognition task. Color indicates the percentage of each data set exhibiting a specific type of error for each word recognition trial (red indicates 50% of cases). Note that the gaps at the beginning, middle, and end of the tasks indicate resting trials when words were not presented (Data Set 1: seven quiet and then seven noise rest trials before and after each word recognition block; Data Set 2: 12 quiet rest trials and then six noise rest trials before each word recognition block, followed by 10 quiet rest trials).

Figure 2 presents the pattern of word recognition in noise errors for each data set, which shows that more initial and final consonant errors were observed compared to vowel errors, whole word omissions, and additive phoneme errors. While this pattern of results was similar in both data sets, there were some differences between data sets. For example, participants in Data Set 1 produced a greater proportion of whole word omission and whole word substitution errors than those in Data Set 2, whereas initial consonant errors were more common in Data Set 2 than Data Set 1, as expected for an open-set word recognition task compared to a voicing task limited to /b/ and /p/ initial consonant words, respectively (Table 2). Supplementary Table S3 presents the mean word recognition scores by listening conditions for each data set.

**Table 2.** Proportion of each error type relative to other word recognition errors across data sets.

	Data Set 1		Data Set 2		Difference estimates	
	*M*	*SD*	*M*	*SD*	*t*	*p*
Average no. errors	32.28	8.13	36.47	12.34	−1.82	0.072
**Initial consonant**	**27.09**	**7.77**	**39.92**	**14.84**	**4.90**	**<0.001**
Final consonant	33.18	10.37	31.80	13.93	−0.51	0.611
**Whole word omission**	**15.54**	**15.24**	**6.28**	**10.17**	**−3.33**	**0.001**
**Whole word substitution**	**8.27**	**5.97**	**1.61**	**2.34**	**−6.91**	**<0.001**
Additive phoneme	7.34	7.71	5.41	6.30	−1.28	0.205
**Vowel**	**8.58**	**5.41**	**14.98**	**7.49**	**4.66**	**<0.001**

*Note*. Errors sum to 100% because the frequency of each error type was computed relative to each of the other error types. **Bold** text indicates factors that were significant predictors in both data sets after Bonferroni correction (*p* = 0.05/6 comparisons).

#### Individual differences

Demographic (age, sex, education) and hearing sensitivity (better ear PTA) variables were not significantly associated with overall word recognition accuracy or the proportion of each error type across both data sets (Table 3). Overall word recognition accuracy was consistently better in participants with faster cognitive processing speed (Connections Complex) in both data sets (Data Set 1: *rho*_38_ = 0.352, *p* = 0.026; Data Set 2: *rho*_43_ = 0.412, *p* = 0.005). Processing speed was not significantly associated with a specific type of error across participants, and thus, no additional processing speed results are presented here.

**Table 3.** Spearman correlation (rho) coefficients representing the associations between overall word recognition accuracy and the proportion of each error type with demographic and cognitive variables.

	Overall accuracy	Initial consonant error	Final consonant error	Whole word omission	Whole word substitution error	Additive phoneme error	Vowel error
**Data Set 1 (*n* = 41)**							
Overall accuracy	1.000	0.092	0.147	−0.136	−0.098	−0.233	−0.119
Age	−0.022	0.044	0.125	−0.054	0.044	−0.170	0.036
Sex (1: male; 2: female)	0.132	0.164	0.320	−0.055	0.068	−0.725	−0.023
Education	0.043	0.051	−0.033	−0.270	0.184	0.326	−0.012
Better Ear PTA (db HL)	−0.152	0.199	−0.077	0.111	−0.118	−0.040	0.082
CPT omissions	0.042	−0.185	0.165	−0.012	−0.123	0.019	0.008
CPT commissions	0.309	0.047	0.122	−0.034	−0.072	−0.016	0.070
CNX speed (simple)	0.261	0.315	−0.101	−0.074	−0.002	−0.153	0.067
**CNX speed (complex)**	**0.352**	0.173	−0.160	−0.021	−0.052	−0.211	−0.048
Stroop inhibitory control	−0.111	0.206	−0.298	−0.078	0.260	−0.025	−0.127
WCST total	0.313	0.124	−0.225	0.137	−0.133	0.165	−0.250
WISC vocabulary	0.059	0.007	−0.171	0.017	−0.144	0.445	−0.013

**Data Set 2 (*n* = 45)**							
Overall accuracy	1.000	−0.350	0.443	−0.174	−0.104	0.324	−0.092
Age	−0.131	0.117	−0.035	−0.125	0.274	−0.156	0.059
Sex (1: male; 2: female)	0.421	−0.123	0.130	−0.093	−0.206	0.255	0.156
Education	0.086	0.178	−0.050	−0.155	−0.176	0.012	0.096
PTA (better ear)	−0.404	−0.219	0.106	0.092	0.244	−0.024	0.144
CPT omissions	−0.058	0.283	−0.374	0.331	0.002	−0.090	0.183
CPT commissions	−0.087	0.139	−0.084	0.140	−0.241	−0.149	0.035
CNX speed (simple)	0.483	−0.506	0.412	−0.111	−0.144	0.289	0.092
**CNX speed (complex)**	**0.412**	−0.468	0.501	−0.151	−0.007	0.176	0.014
Stroop inhibitory control	0.310	−0.419	0.326	−0.008	−0.213	0.390	−0.131
WCST total	0.410	−0.170	0.126	−0.062	−0.053	0.052	−0.177
WAIS vocabulary	0.215	−0.415	0.306	−0.173	−0.168	0.117	0.273

*Note*. Significant effects in both data sets, *p* < 0.05, as indicated by **bold** text. Proportion of each error type is relative to the sum of each of the error types listed to present their relative associations with the demographic and cognitive variables. Similar nonsignificant results were observed for the sum of each error type, with the exception that overall accuracy was significantly related to initial consonant errors (Data Set 1: *r* = −0.548, *p* < 0.001; Data Set 2: *r* = −0.730, *p* < 0.001). PTA = better ear pure-tone average (dB HL; average of thresholds from 0.25 to 8 kHz); CPT = Conners Continuous Performance Test; CNX = Connections Cognitive Processing Speed Test; WCST = Wisconsin Card Sorting Task; WISC = Wechsler Adult Intelligence Scales.

### Cingulo-Opercular and Auditory Network Activity Relate to Word Recognition Errors

**Figure 3.**
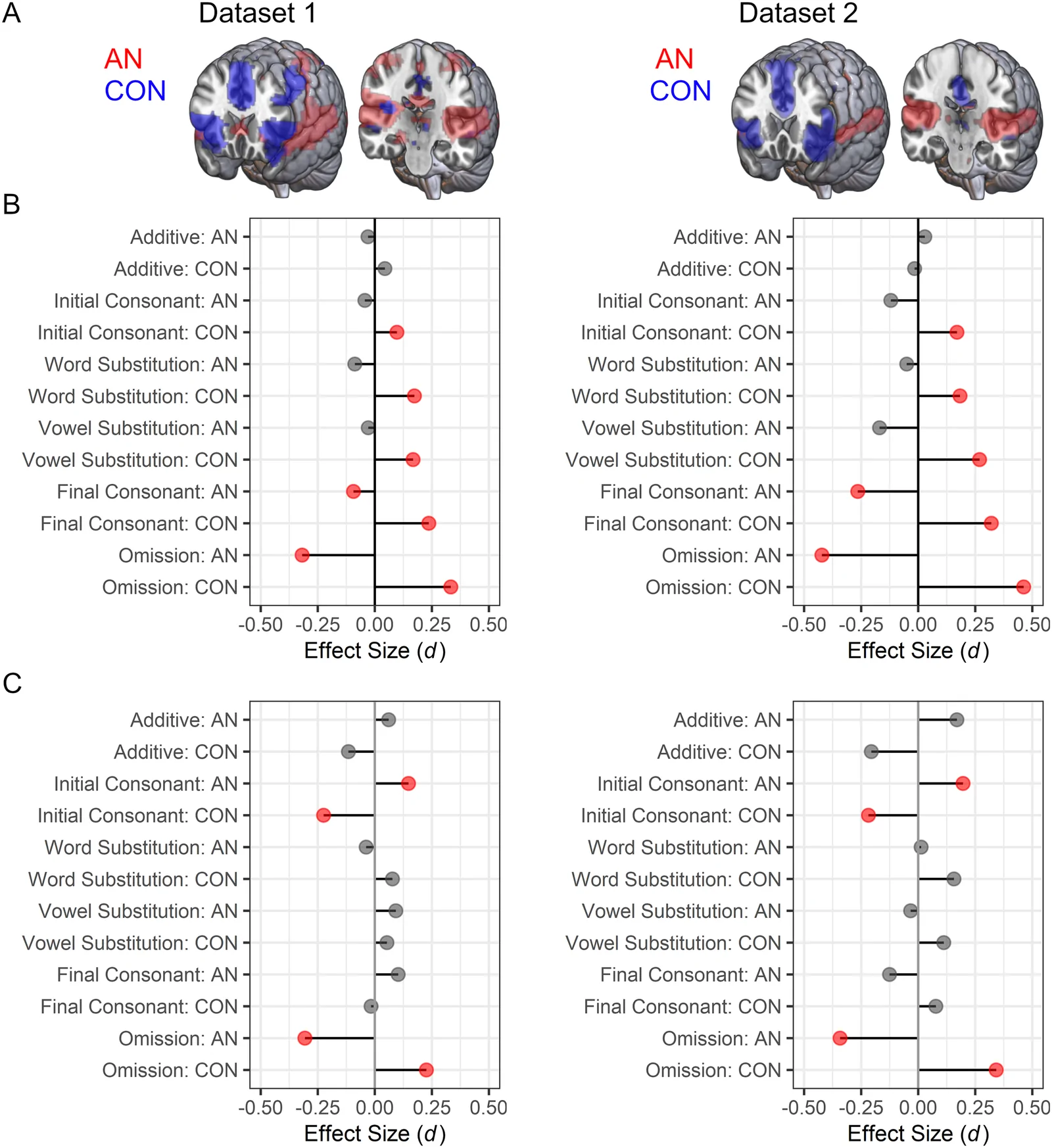
Word recognition error types were associated with distinct patterns of auditory network (AN) and cingulo-opercular network (CON) activity. (A) Spatial distribution of the networks (family-wise error corrected *p* < 0.05). (B, C) Cohen’s *d* effect sizes are shown for error type effects for comparisons of each error type and correct trials (B) or each error type compared to every other error (C). Red symbols indicate significant effects or associations with *p* values < 0.0083 in both data sets. Additive errors occurred when a phoneme was added to the beginning or end of the presented word. Initial errors occurred when only the initial consonant of the word was incorrect. Vowel errors were observed when the vowel sound was reported incorrectly. Omission errors occurred when the participant could not understand the word and said “nope.” The additive error and CON association in plot C approached but did not exhibit a significant effect across both data sets (Data Set 1: *p* = 0.018; Data Set 2: *p* < 0.001).

The ICA GIFT analyses identified 48 and 38 distinct patterns of activity that explained 94.01% and 93.42% of the brain activity variance in Data Set 1 and Data Set 2, respectively. These independent components included spatial patterns of activity that correspond to auditory and cingulo-opercular networks (Figure 3A). There was a high degree of spatial overlap for each network across data sets, as demonstrated by pairwise correlation of the binarized voxels of the corresponding network images (auditory: *r*_7221032_ = 0.520, *p* < 0.001; cingulo-opercular: *r*_7221032_ = 0.514, *p* < 0.001).

#### Differences in activity for each error type relative to correct trials

Lower auditory network activity was significantly associated with whole word omission errors and final consonant errors compared to correct trials (Figure 3B). Elevated cingulo-opercular activity was also significantly associated with whole word omissions, whole word substitutions, initial consonant, final consonant, and vowel errors compared to correct trials, but not additive phoneme errors.

#### Differences in activity for each error type relative to all other errors

Lower auditory network activity was significantly associated with whole word omission errors compared to all other error trials, whereas higher auditory network activity was associated with initial consonant errors compared to all other errors (Figure 3C). Higher cingulo-opercular activity was also significantly associated with whole word omission errors compared to all other error types (Figure 3C). In contrast, initial consonant phoneme errors were significantly associated with lower cingulo-opercular activity compared to all other errors. Together, these results show that auditory network activity was relatively low and cingulo-opercular activity was relatively high when participants failed to generate their own word recognition response or made a final consonant error. In contrast, misperception of the initial consonant occurred with a level of auditory network activity that was not substantively different from the auditory network activity during correct responses. Supplementary Tables S4 and S5 present the parameter estimates for the results shown in Figure 3.

The results from these error analyses also suggested that cortical network activity relates to the type of error rather than the extent of error, as demonstrated when comparing error types with the same number of missed phonemes. Specifically, final consonant errors exhibited more cingulo-opercular activity compared to initial consonant errors in Data Set 1 (cingulo-opercular network: *t*(723) = 2.895, *p* = 0.004; auditory network: *t*(723) = −0.733, *p* = 0.464) and Data Set 2 (cingulo-opercular network: *t*(911) = 3.201, *p* = 0.001; auditory network: *t*(900) = −3.467, *p* < 0.001). That is, final and initial consonant errors with the same number of missed phonemes exhibited significant differences in cingulo-opercular activity, thereby indicating that the type and not extent of error is related to cingulo-opercular activity.

### DMN Activity Relates to Omissions

**Figure 4.**
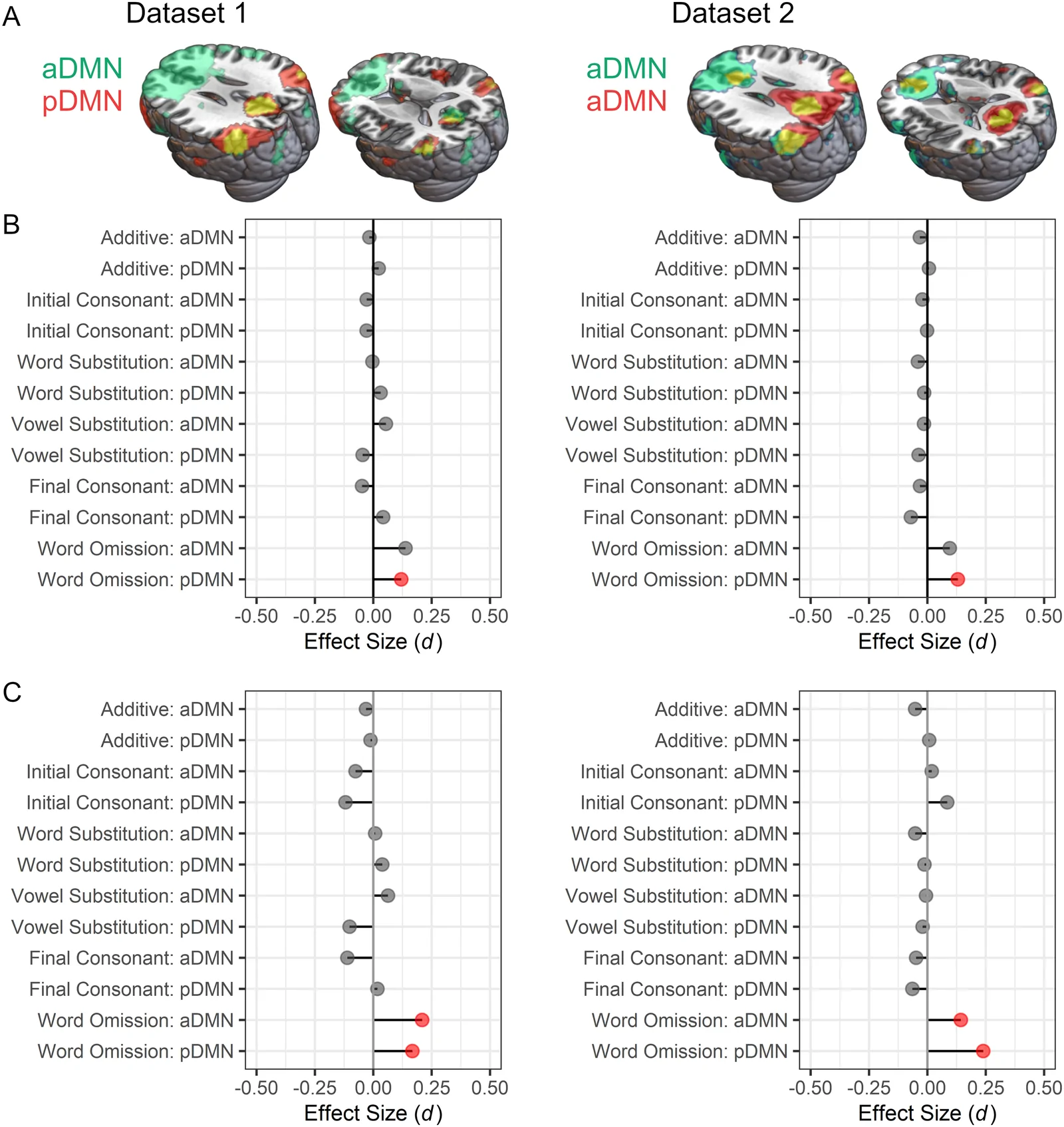
Elevated DMN activity was associated with omission errors. (A) Distinct patterns of anterior and posterior DMN activity (Kim & Lee, 2011; Liu et al., 2022; Taylor et al., 2023). Yellow in the rendering indicates overlap of the aDMN (green) and pDMN (orange). Elevated DMN activity was significantly related to omission errors compared to (B) correct trials and (C) all other error trials. Cohen’s *d* effect sizes are shown with red symbols indicating significant effects or associations with *p* values < 0.0083 in both data sets.

Two patterns of DMN activity were observed in both data sets and appeared to correspond to anterior and posterior spatial distributions of the DMN, where one network in each data set included more ventromedial prefrontal cortex and the other network included more precuneus and bilateral parietal cortex, respectively. These two patterns of DMN activity exhibited significant spatial overlap between data sets (anterior DMN: *r*_7221032_ = 0.578, *p* < 0.001; posterior DMN: *r*_7221032_ = 0.355, *p* < 0.001), as shown in Figure 4A. GLMM showed that elevated posterior DMN activity was significantly associated with whole word omission errors compared to correct trials (Figure 4B). Elevated posterior and anterior DMN activity also predicted a higher likelihood of whole word omissions when compared to all other error trials (Figure 4C). There were no significant DMN associations with other error types across data sets, as shown in Supplementary Tables S6 and S7.

Follow-up GLMM analyses were also performed to assess whether auditory and cingulo-opercular network activity could explain the association between posterior DMN activity and whole word omission. That is, lower auditory network activity or lower cingulo-opercular activity might be expected to occur with an increase in DMN activity and thus explain the DMN associations with whole word omissions. Table 4 shows that activity within default mode, auditory, and cingulo-opercular networks predicted omissions when included in the same GLMM. These results suggest that varied combinations of decreased auditory network activity, increased cingulo-opercular, and/or increased DMN activity are associated with omissions when middle-aged to older adults perform speech recognition in noise tasks.

**Table 4.** GLMM results showing that whole word omissions compared to correct or other incorrect trials occur with elevated DMN activity, lower auditory network activity, and higher cingulo-opercular activity.

		Estimate	*SE*	*df*	*t*	*p*
Omissions vs. correct trials						
Data Set 1	Anterior DMN	0.021	0.005	3729	4.412	0.000
	Posterior DMN	0.005	0.004	3731	1.256	0.209
	**Auditory network**	**−0.047**	**0.005**	**3729**	**−9.908**	**0.000**
	**Cingulo-opercular network**	**0.043**	**0.005**	**3730**	**9.281**	**0.000**
Data Set 2	Anterior DMN	−0.007	0.005	2859	−1.377	0.169
	Posterior DMN	0.024	0.004	2860	5.432	0.000
	**Auditory network**	**−0.064**	**0.005**	**2859**	**−11.718**	**0.000**
	**Cingulo-opercular network**	**0.069**	**0.006**	**2863**	**12.232**	**0.000**

Omissions vs. incorrect trials						
Data Set 1	Anterior DMN	0.045	0.010	1651	4.762	0.000
	Posterior DMN	0.022	0.010	1659	2.257	0.024
	**Auditory network**	**−0.064**	**0.010**	**1651**	**−6.536**	**0.000**
	**Cingulo-opercular network**	**0.036**	**0.010**	**1657**	**3.772**	**0.000**
Data Set 2	Anterior DMN	0.006	0.009	1573	0.637	0.524
	Posterior DMN	0.041	0.008	1570	5.427	0.000
	**Auditory network**	**−0.062**	**0.009**	**1577**	**−6.961**	**0.000**
	**Cingulo-opercular network**	**0.059**	**0.009**	**1593**	**6.334**	**0.000**

*Note*. **Bold** text indicates factors that were significant predictors in both data sets. GLMM = generalized linear mixed model; DMN = default mode network.

## DISCUSSION

Distinct types of word recognition errors occurred with different patterns of cortical network activity. Whole word omissions were significantly more likely with lower auditory network activity and/or elevated default mode activity. One consequence of these errors appears to be an elevation of cingulo-opercular network activity that is consistent with the premise that missing more speech sounds of a word produces a larger frontal performance monitoring response. In addition, final consonant and initial consonant substitution errors do not differ in the extent of error (i.e., one missed phoneme), but lower auditory network activity and higher cingulo-opercular activity were observed for final consonant errors compared to initial consonant errors. Together, these findings support the premise that neural activity differs by error type and suggest that there are different neural mechanisms and consequences for these errors.

### Patterns of Errors

The different types of word recognition errors observed in this study are consistent with evidence that listeners are more likely to misperceive the final consonant than the initial consonant during real word and non-word recognition tasks (Dubno et al., 1982; Miller & Nicely, 1955; Oyer & Doudna, 1959). There were differences in the proportion of different error types between data sets in the current study that appeared to depend on whether the task involved open-set or relatively closed-set word recognition. Final consonant errors were more common than initial consonant errors in the open-set word recognition experiment, whereas initial consonant errors were more common in the relatively more closed-set /b/ and /p/ initial consonant task. Different initial consonant sounds that vary in voicing but have the same manner of articulation may be more likely to be misperceived (e.g., /b/ and /p/ consonants) than initial consonants with different manners of articulation (e.g., /b/ and /sh/). Word lists limited to the same manner of articulation appear to shift the pattern of word recognition errors. The differences in the frequency of initial and final consonant errors in each data set were modest, however, and initial and final consonant errors were observed more frequently compared to additive, omission, or vowel errors across both data sets.

### Cognitive Effects

Faster cognitive processing speed was associated with better overall word recognition accuracy in both data sets, and these results replicate prior evidence that faster processing speed predicts better word recognition in noise across studies with different experimental designs (Dryden et al., 2017). However, we found no consistent associations between cognitive measures and any particular type of error across data sets. There were trends toward statistically significant effects, such as a vocabulary knowledge association with the frequency of adding a speech sound to a target word. Together, these results suggest (1) that the experimental design (open set with varying SNR conditions vs. relatively closed set with voicing and pitch conditions) influenced the distribution of error types and (2) that middle-aged to older adults with slower (vs. faster) processing speed are expected to make more errors across experimental designs, regardless of the relative frequency of each error type, which may be why cognitive processing speed is consistently related to differences in word recognition across studies.

### Network Activity During Word Recognition in Noise

The patterns of brain activity observed in this study are consistent with previous studies of speech recognition in noise that showed elevated auditory network activity with increased speech intelligibility and speech understanding (Evans & McGettigan, 2017) and elevated cingulo-opercular cortex activity with decreased speech intelligibility (Alain et al., 2018; Eckert et al., 2016). These cingulo-opercular effects may stem from the increased likelihood for word recognition error, task difficulty, and response uncertainty (Clairis & Lopez-Persem, 2023), particularly when the task is difficult but participants are still able to accurately perform the task (Kuchinsky & Vaden, 2020). This idea is consistent with evidence that participant ratings of increased trial-level task difficulty relate to increased cingulo-opercular activity (Desender et al., 2021).

The results of this study also demonstrate that DMN activity was higher when listeners made omission errors relative to correct responses and other error types. Elevated DMN activity has been observed with increasing speech intelligibility (Eckert et al., 2008; Obleser et al., 2007). This pattern of activity can reflect semantic processing (Wirth et al., 2011) and anticipation of meaning for fragmented or partial speech stimuli (Scharinger et al., 2016), and/or construction of situational or overall task experience (Fernandino & Binder, 2024). However, semantic networks that are spatially distinct from the DMN have been observed (Rysop et al., 2025), and DMN activity during speech tasks may also reflect lower task demand (Humphreys et al., 2015) and/or mind-wandering (Kucyi et al., 2016). Together, these results suggest that elevated DMN activity during single word recognition tasks with no semantic analysis demands can indicate task disengagement that increases the likelihood of word omission errors. This effect may be more likely when speech is intelligible compared to when listeners have to work to understand speech, perhaps unless speech is sufficiently unintelligible that listeners know that they cannot understand it and subsequently disengage from the task (i.e., a U-shaped distribution of DMN activity across a range of floor-to-ceiling task performance).

### Initial and Additive Phoneme Errors

Initial and additive phoneme errors occurred with just as much auditory network activity as words that were correctly recognized. Initial phoneme errors also occurred with less cingulo-opercular activity compared to all other errors (with a similar pattern of results for additive errors; Figure 3), suggesting the possibility that listeners did not recognize their error during tasks where performance feedback was not provided. This subjective listening confidence or metacognition interpretation (Obleser, 2025) would be strengthened by participant report about whether they understood each item and provides an empirical foundation for testing the hypothesis that listener over-confidence during speech recognition (Bernstein et al., 2021) can be explained by a pattern of elevated auditory network activity and lower cingulo-opercular activity.

These results also suggest that the magnitude of auditory network activity is not sufficient to explain why listeners demonstrate initial and additive phoneme errors. Background noise and/or low-pass filtering of the speech could disrupt accurate voicing perception or contribute to the perception of an additional phoneme, respectively. It is also possible that speech cues were miscoded at earlier stages in the auditory pathway because of peripheral and/or subcortical factors (Anderson et al., 2018; Harris et al., 2021), particularly when words had shorter voice onset times. This miscoding would be propagated to the auditory cortex, thereby resulting in misperception but not differences in the magnitude of auditory cortex activity. Given a positive association between fMRI contrast and high-frequency electrocorticography (ECoG) measures (Haufe et al., 2018), the miscoding explanation could be examined using ECoG local field power and spectrotemporal tuning metrics (Leonard et al., 2024). Here, overall local field power may not differ between correct and incorrect perception, but it may be possible to see differential spectrotemporal tuning for correct and incorrect perception.

### Whole Word Omission Errors

Complete word omission errors exhibited the largest error type effects across auditory, default mode, and cingulo-opercular networks. The lower auditory network activity during omission errors suggests that insufficient signal reached auditory cortex to reconstruct a degraded speech stimulus. These errors also occurred with elevated DMN activity that suggested mind-wandering or disengagement from the task during these trials. That is, the results suggest two independent explanations for why a listener did not generate their own response: They were not able to extract the target word from noise and/or they were not attending to the speech.

The inability to generate a word recognition response occurred with a large cingulo-opercular response compared to correct and other incorrect trials, at least when participants were sufficiently engaged in the task to produce a “nope” response. Cingulo-opercular cortex is important for performance monitoring to support adaptive behavior when there is expected value (Eckert et al., 2016; Pichora-Fuller et al., 2016; Shenhav et al., 2016). Thus, this large response for omission trials may facilitate relatively large changes in behavior and attentional state (i.e., increased tonic alertness [Sadaghiani & D’Esposito, 2015] and/or more cautious responding [Vaden et al., 2022]).

### Final Consonant Errors

The final consonant error results are consistent with the premise examined in this study that the type of word recognition error, and not specifically the extent of error, would relate to the magnitude of cingulo-opercular activity. Final consonant errors occurred with higher cingulo-opercular activity in comparison to the initial consonant errors, for example (Figure 3C). While additional study is necessary, the final consonant errors appear to contribute more to the overall error-related cingulo-opercular response in speech-in-noise recognition tasks (Alain et al., 2018; Eckert et al., 2016; Peelle, 2018), compared to other single phoneme errors. Moreover, cingulo-opercular cortex may track final consonant responses to a greater extent than initial consonant responses, for example, because final consonant errors are one of the most common types of errors in word and pseudoword recognition studies (Dubno et al., 1982; Miller & Nicely, 1955). It is also possible that cingulo-opercular cortex is sensitive to final consonant errors because the final consonant is critical for differentiating minimal word pairs with common onset syllables, which are relatively more frequent across languages than minimal pairs with common coda syllables (Sun & Poeppel, 2023).

To the extent that the cingulo-opercular pattern of network activity in this study reflects an action mode network for goal-directed interaction with the environment (Dosenbach et al., 2025), these final consonant errors would be expected to produce more adaptive behavior during word recognition. In addition to changes in alertness and cautious responding, we hypothesize that participants are more likely to adjust their behavior to optimize speech understanding following a final consonant error. For example, adjustments may include closing one’s eyes (Mohanathas et al., 2024), head tilting toward a sound source (Minagi et al., 2025), and slowing response selection, which may increase word recognition accuracy in some listening conditions (Vaden et al., 2022).

### Limitations and Future Directions

The data examined in this study were from single word recognition tasks. This reduced complexity of results interpretation compared to connected speech where there are additional higher-level language and working memory demands (Peelle, 2018). Word recognition with limited comprehension and working memory demands also limited study of other networks that appear to support speech recognition tasks with working memory demands. For example, frontoparietal cortex exhibits elevated activity when the speech recognition task involves continuous speech and presumably requires working memory (Meyer et al., 2012) to track and understand the temporally unfolding semantic (Golestani et al., 2013) and syntactic content (Peelle, Troiani, et al., 2010), particularly with an increasing rate of stimulus presentation (Peelle et al., 2004). That is, the current results cannot address questions about the extent to which low frontoparietal activity occurs with omissions because of difficulty recalling all of the words in a sentence.

The two data sets examined in this study had different task designs, differed by lexical features, and included middle-aged to older adults who were not matched for demographic and cognitive profiles. This could have impacted the likelihood of replication across data sets. For example, lower auditory network activity occurred with vowel errors in Data Set 2 (*p* < 0.001), but not Data Set 1 (*p* = 0.386). In addition, there were relatively few trials with vowel or whole word substitution errors. In spite of this, there was consistency in the direction and size of effects across the two data sets, in combination with multiple comparison correction within each data set. This suggests generalizability of the results across word recognition in noise studies with varied experimental features (e.g., phonological neighborhood density). This replication approach also helped to address concerns about the relatively low frequency of whole word omission errors compared to other types of errors, including because the omission errors produced some of the largest network activity associations. That is, there were large effect sizes for the whole word omission errors that were reliably observable in this study, but future studies may need targeted experimental manipulations to examine brain activity associated with whole word substitution and vowel errors to ensure adequate power.

There was clear spatial overlap between data sets for each of the auditory, cingulo-opercular, and DMNs, although to a lesser extent for the posterior DMN. While anterior and posterior DMN activity predicted omission errors relative to correct trials in both data sets, the inclusion of the cingulo-opercular and auditory networks in the regression models differentially affected anterior and posterior DMN associations with omissions across data sets. DMN activity is often suppressed during challenging tasks (Menon, 2023) and can be influenced by ongoing task demands (Sormaz et al., 2018). Thus, differences in DMN patterns of activity between data sets and associations with omission errors could be due to differences in the open-set and relatively closed-set word recognition tasks. DMN activity has also been associated with individual differences, including demographic variables like age (Malagurski et al., 2022; Mevel et al., 2013). Data Set 1 participants were older than participants in Data Set 2. While this ∼4 year mean age difference was modest, differential DMN aging between data sets could have contributed to the DMN results, and thus, anterior relative to posterior DMN associations with word recognition errors require future study.

### Clinical Relevance

A single speech recognition-in-noise score provides an index of ability that is useful for evaluating overall performance relative to the broader population but does not always provide insight into why listeners have speech recognition difficulty. The results of this study suggest that listeners who do not generate a word recognition response either have an insufficient signal in the auditory cortex and/or engage DMN functions and are insufficiently attentive during those recognition errors. While additional study is needed, the results also suggest that listeners making an initial consonant error or an additive phoneme error are less aware of the error compared to a final consonant error based on relatively lower auditory network activity and higher cingulo-opercular activity observed for final consonant errors. That is, listeners with similar speech recognition in noise scores may have different neurobiological explanations for their errors that can be inferred from the types of errors that they make. These results also suggest that the types of errors that listeners make may influence listening effort that occurs when listeners work to repair misperceptions (Smith & Winn, 2025).

### Conclusions

The results of the current study demonstrate that (1) the magnitude of auditory network activity is not significantly different between correct trials and trials with initial consonant and additive phoneme errors; (2) relatively large performance monitoring responses are related to final consonant and whole word omission errors; and (3) word omissions occur when auditory network activity is low, cingulo-opercular activity is high, and/or DMN activity is high. Thus, patterns of brain activity differ for different types of word recognition errors that contribute to an overall word recognition score among middle-aged to older adult listeners. These results show that examination of the types of word recognition error can inform reason(s) for those errors that may not be clearly evident in an overall word recognition score.

## ACKNOWLEDGMENTS

We thank the participants for their contributions to this project.

## FUNDING INFORMATION

Judy R. Dubno, National Institute on Deafness and Other Communication Disorders (https://dx.doi.org/10.13039/100000055), Award ID: P50DC000422. National Center for Research Resources, Award ID: UL1TR001450. National Center for Research Resources, Award ID: C06RR14516.

## AUTHOR CONTRIBUTIONS

**Mark A. Eckert**: Conceptualization; Data curation; Formal analysis; Funding acquisition; Investigation; Methodology; Resources; Software; Visualization; Writing – original draft; Writing – review & editing. **Kenneth I. Vaden**: Conceptualization; Investigation; Writing – review & editing. **Carolyn M. McClaskey**: Conceptualization; Investigation; Writing – review & editing. **Susan Teubner-Rhodes**: Conceptualization; Investigation; Writing – review & editing. **Judy R. Dubno**: Conceptualization; Funding acquisition; Resources; Writing – review & editing.

## DATA AND CODE AVAILABILITY STATEMENT

The de-identified primary data used in this study are available upon request and completion of institutional data use agreements, as required by the Medical University of South Carolina. The data analysis R code and data to generate the results presented here are available at https://osf.io/7xmrp.

## Supplementary Material


