# Supplementary material for: Cortical Network Activity Associated With Different Types of Word Recognition Errors by Older Adults
Source: Neurobiol Lang (Camb). 2026 Jul 28;7:NOL.a.275. doi: 10.1162/NOL.a.275 (PMC13597106; doi:10.1162/NOL.a.275)
Supplement: Supplementary file 1 [file nol-07-275-s001.docx]

**Supplementary Data**

Supplementary Table 1. Dataset 1 words that were presented during the fMRI word recognition in noise task.

| bath | fall | home | long | near | rode | thin | wire |
| --- | --- | --- | --- | --- | --- | --- | --- |
| beg | food | jail | lore | neat | room | thought | young |
| boat | gap | jug | love | note | rose | thumb |  |
| bought | germ | kill | luck | pain | said | time |  |
| cab | get | knock | make | perch | sell | tire |  |
| cool | goal | late | match | phone | shawl | tough |  |
| date | good | lean | merge | pool | soup | void |  |
| dip | gun | learn | mill | rain | south | wag |  |
| dodge | hate | life | moon | rat | take | walk |  |
| door | have | live | nag | red | tell | wife |  |

Supplementary Table 2. Dataset 2 words that were presented during the fMRI word recognition in noise task.

| back | beep | boys | pair | poise | push |
| --- | --- | --- | --- | --- | --- |
| bad | bees | bro | pale | pole | puss |
| bale | big | bull | par | pop |  |
| bane | birch | bun | pass | pose |  |
| bar | boast | bus | peach | post |  |
| base | bop | bush | peak | pot |  |
| bass | bore | pace | peas | pour |  |
| beach | bought | pack | peep | pro |  |
| beak | bowl | pad | perch | pull |  |
| bear | bows | pain | pig | pun |  |

Supplementary Table 3. The average percent correct performance is presented for each dataset by listening conditions.

| Dataset 1 |  |  | Dataset 2 |  |  |
| --- | --- | --- | --- | --- | --- |
| Condition | Mean | SD | Condition | Mean | SD |
| 2 dB SNR: Cued | 56.32 | 9.29 | B Words: High Pitch | 50.73 | 17.46 |
| 2 dB SNR: Uncued | 57.13 | 11.47 | B Words: Low Pitch | 57.56 | 15.91 |
| 10 dB SNR: Cued | 76.10 | 10.30 | P Words: High Pitch | 75.52 | 12.09 |
| 10 dB SNR: Uncued | 79.41 | 9.49 | P Words: Low Pitch | 72.62 | 12.65 |

Cued: A visual symbol was presented to indicate the lower or high signal-to-noise ratio (SNR). Uncued: An uninformative cue was presented. B words: B-initial consonant words; P Words: P-initial consonant words

Supplementary Table 4. Auditory network parameter estimates from the GLMM analyses where the auditory and cingulo-opercular network variables were included as predictors of each error type relative to correct trials or relative to all other error types.

| Auditory Network | | | | | | | |
| --- | --- | --- | --- | --- | --- | --- | --- |
|  |  | Error Type | *Estimate* | *SE* | *df* | *t* | *p* |
| Dataset 1: |  | Initial Consonant | -0.008 | 0.006 | 3856.790 | -1.355 | 0.176 |
| Error Type vs Correct Trials |  | **Final Consonant** | **-0.018** | **0.006** | **3925.797** | **-2.931** | **0.003** |
|  |  | **Omission** | **-0.045** | **0.005** | **3730.331** | **-9.730** | **0.000** |
|  |  | Additive | -0.003 | 0.003 | 3612.623 | -0.898 | 0.370 |
|  |  | Vowel | -0.003 | 0.004 | 3631.595 | -0.866 | 0.386 |
|  |  | Word Substitution | -0.009 | 0.004 | 3629.067 | -2.642 | 0.008 |
| Dataset 2: |  | Initial Consonant | -0.039 | 0.011 | 3309.933 | -3.448 | 0.001 |
| Error Type vs Correct Trials |  | **Final Consonant** | **-0.076** | **0.010** | **3155.352** | **-7.440** | **0.000** |
|  |  | **Omission** | **-0.061** | **0.005** | **2860.605** | **-11.288** | **0.000** |
|  |  | Additive | 0.004 | 0.005 | 2851.952 | 0.776 | 0.438 |
|  |  | Vowel | -0.037 | 0.008 | 2979.208 | -4.625 | 0.000 |
|  |  | Word Substitution | -0.004 | 0.003 | 2816.665 | -1.336 | 0.182 |
| Dataset 1: |  | **Initial Consonant** | **0.037** | **0.012** | **1684.000** | **3.029** | **0.002** |
| Error Type vs All Other Error Trials |  | Final Consonant | 0.028 | 0.013 | 1677.470 | 2.098 | 0.036 |
|  |  | **Omission** | **-0.060** | **0.010** | **1652.467** | **-6.229** | **0.000** |
|  |  | Additive | 0.008 | 0.007 | 1676.151 | 1.243 | 0.214 |
|  |  | Vowel | 0.014 | 0.007 | 1684.000 | 1.891 | 0.059 |
|  |  | Word Substitution | -0.006 | 0.009 | 1465.796 | -0.727 | 0.467 |
| Dataset 2: |  | **Initial Consonant** | **0.081** | **0.022** | **1415.221** | **3.698** | **0.000** |
| Error Type vs All Other Error Trials |  | Final Consonant Error | -0.048 | 0.020 | 1417.964 | -2.365 | 0.018 |
|  |  | **Omission** | **-0.061** | **0.009** | **1579.290** | **-6.807** | **0.000** |
|  |  | Additive | 0.028 | 0.008 | 1591.927 | 3.386 | 0.001 |
|  |  | Vowel | -0.010 | 0.015 | 1425.975 | -0.624 | 0.532 |
|  |  | Word Substitution | 0.001 | 0.005 | 1425.233 | 0.241 | 0.810 |

Bold text indicates factors that were significant predictors in both datasets.

Supplementary Table 5. Cingulo-opercular network parameter estimates from GLMM analyses where the auditory and cingulo-opercular network variables were predictors of each error type relative to correct trials or relative to all other error types.

| Cingulo-opercular Network | | | | | | | |
| --- | --- | --- | --- | --- | --- | --- | --- |
|  |  | Error Type | *Estimate* | *SE* | *df* | *t* | *p* |
| Dataset 1: |  | **Initial Consonant** | **0.018** | **0.006** | **3864.545** | **3.012** | **0.003** |
| Error Type vs Correct Trials |  | **Final Consonant** | **0.045** | **0.006** | **3926.355** | **7.415** | **0.000** |
|  |  | **Omission** | **0.047** | **0.005** | **3731.681** | **10.178** | **0.000** |
|  |  | Additive | 0.004 | 0.003 | 3616.582 | 1.330 | 0.183 |
|  |  | **Vowel** | **0.018** | **0.004** | **3635.478** | **5.054** | **0.000** |
|  |  | **Word Substitution** | **0.019** | **0.004** | **3632.833** | **5.229** | **0.000** |
| Dataset 2: |  | **Initial Consonant** | **0.052** | **0.011** | **3312.691** | **4.890** | **0.000** |
| Error Type vs Correct Trials |  | **Final Consonant** | **0.086** | **0.010** | **3163.487** | **9.015** | **0.000** |
|  |  | **Omission** | **0.064** | **0.005** | **2864.278** | **12.370** | **0.000** |
|  |  | Additive | -0.002 | 0.005 | 2859.984 | -0.420 | 0.675 |
|  |  | **Vowel** | **0.055** | **0.007** | **2984.322** | **7.348** | **0.000** |
|  |  | **Word Substitution** | **0.013** | **0.003** | **2820.864** | **4.867** | **0.000** |
| Dataset 1: |  | **Initial Consonant** | **-0.055** | **0.012** | **1684.000** | **-4.607** | **0.000** |
| Error Type vs All Other Error Trials |  | Final Consonant | -0.004 | 0.013 | 1683.459 | -0.337 | 0.736 |
|  |  | **Omission** | **0.043** | **0.009** | **1660.209** | **4.605** | **0.000** |
|  |  | Additive | -0.016 | 0.007 | 1690.025 | -2.378 | 0.018 |
|  |  | Vowel | 0.008 | 0.007 | 1684.000 | 1.090 | 0.276 |
|  |  | Word Substitution | 0.012 | 0.008 | 1473.769 | 1.484 | 0.138 |
| Dataset 2: |  | **Initial Consonant** | **-0.082** | **0.020** | **1425.965** | **-4.120** | **0.000** |
| Error Type vs All Other Error Trials |  | Final Consonant | 0.027 | 0.018 | 1425.604 | 1.462 | 0.144 |
|  |  | **Omission** | **0.057** | **0.008** | **1592.695** | **6.823** | **0.000** |
|  |  | Additive | -0.032 | 0.008 | 1602.987 | -4.114 | 0.000 |
|  |  | Vowel | 0.029 | 0.014 | 1393.539 | 2.091 | 0.037 |
|  |  | Word Substitution | 0.014 | 0.005 | 1380.334 | 2.911 | 0.004 |

Bold text indicates factors that were significant predictors in both datasets.

Supplementary Table 6. Anterior default mode network parameter estimates from the GLMM analyses where the anterior and posterior network variables were predictors of each error type relative to correct trials or relative to all other error types.

| Anterior Default Mode Network | | | | | | | |
| --- | --- | --- | --- | --- | --- | --- | --- |
|  |  | Error Type | *Estimate* | *SE* | *df* | *t* | *p* |
| Dataset 1: |  | Initial Consonant | -0.005 | 0.006 | 3867 | -0.869 | 0.385 |
| Error Type vs Correct Trials |  | Final Consonant | -0.009 | 0.006 | 3926 | -1.510 | 0.131 |
|  |  | Omission | 0.020 | 0.005 | 3731 | 4.217 | 0.000 |
|  |  | Additive | -0.002 | 0.003 | 3616 | -0.502 | 0.616 |
|  |  | Vowel | 0.006 | 0.004 | 3637 | 1.631 | 0.103 |
|  |  | Word Substitution | 0.000 | 0.004 | 3633 | -0.111 | 0.911 |
| Dataset 2: |  | Initial Consonant | -0.006 | 0.010 | 3305 | -0.616 | 0.538 |
| Error Type vs Correct Trials |  | Final Consonant | -0.008 | 0.009 | 3160 | -0.851 | 0.395 |
|  |  | Omission ~ | 0.012 | 0.005 | 2861 | 2.565 | 0.010 |
|  |  | Additive | -0.004 | 0.004 | 2848 | -0.861 | 0.390 |
|  |  | Vowel | -0.003 | 0.007 | 2976 | -0.397 | 0.692 |
|  |  | Word Substitution | -0.003 | 0.002 | 2811 | -1.078 | 0.281 |
| Dataset 1: |  | Initial Consonant | -0.019 | 0.012 | 1684 | -1.560 | 0.119 |
| Error Type vs All Other Error Trials |  | Final Consonant | -0.029 | 0.013 | 1680 | -2.279 | 0.023 |
|  |  | **Omission** | **0.040** | **0.009** | **1654** | **4.256** | **0.000** |
|  |  | Additive | -0.004 | 0.007 | 1679 | -0.640 | 0.522 |
|  |  | Vowel | 0.009 | 0.007 | 1684 | 1.294 | 0.196 |
|  |  | Word Substitution | 0.001 | 0.008 | 1468 | 0.155 | 0.877 |
| Dataset 2: |  | Initial Consonant | 0.007 | 0.019 | 1399 | 0.347 | 0.729 |
| Error Type vs All Other Error Trials |  | Final Consonant | -0.015 | 0.018 | 1402 | -0.832 | 0.406 |
|  |  | **Omission** | **0.023** | **0.008** | **1571** | **2.837** | **0.005** |
|  |  | Additive | -0.008 | 0.008 | 1577 | -1.043 | 0.297 |
|  |  | Vowel | -0.002 | 0.014 | 1411 | -0.111 | 0.912 |
|  |  | Word Substitution | -0.005 | 0.005 | 1426 | -0.986 | 0.325 |

~ Did not pass the threshold of a *p* < 0.0083 in both datasets to be defined as a significant effect, but note the consistent direction of effect in both samples. Bold text indicates factors that were significant predictors in both datasets.

Supplementary Table 7. Posterior default mode network parameter estimates from the GLMM analyses where the anterior and posterior network variables were predictors of each error type relative to correct trials or relative to all other error types.

| Posterior Default Mode Network | | | | | | | |
| --- | --- | --- | --- | --- | --- | --- | --- |
|  |  | Error Type | *Estimate* | *SE* | *df* | *t* | *p* |
| Dataset 1: |  | Initial Consonant | -0.005 | 0.005 | 3866 | -0.889 | 0.374 |
| Error Type vs Correct Trials |  | Final Consonant | 0.008 | 0.006 | 3926 | 1.324 | 0.185 |
|  |  | **Omission** | **0.016** | **0.004** | **3732** | **3.656** | **0.000** |
|  |  | Additive | 0.002 | 0.003 | 3618 | 0.705 | 0.481 |
|  |  | Vowel | -0.005 | 0.003 | 3639 | -1.378 | 0.168 |
|  |  | Word Substitution | 0.003 | 0.003 | 3634 | 0.951 | 0.342 |
| Dataset 2: |  | Initial Consonant | 0.000 | 0.009 | 3306 | -0.039 | 0.969 |
| Error Type vs Correct Trials |  | Final Consonant | -0.016 | 0.008 | 3150 | -1.987 | 0.047 |
|  |  | **Omission** | **0.016** | **0.004** | **2861** | **3.484** | **0.001** |
|  |  | Additive | 0.001 | 0.004 | 2845 | 0.177 | 0.860 |
|  |  | Vowel | -0.006 | 0.006 | 2969 | -1.026 | 0.305 |
|  |  | Word Substitution | -0.001 | 0.002 | 2801 | -0.369 | 0.712 |
| Dataset 1: |  | Initial Consonant | -0.030 | 0.012 | 1684 | -2.442 | 0.015 |
| Error Type vs All Other Error Trials |  | Final Consonant | 0.005 | 0.013 | 1682 | 0.365 | 0.715 |
|  |  | **Omission** | **0.033** | **0.010** | **1661** | **3.411** | **0.001** |
|  |  | Additive | -0.002 | 0.007 | 1692 | -0.242 | 0.809 |
|  |  | Vowel | -0.016 | 0.007 | 1684 | -2.085 | 0.037 |
|  |  | Word Substitution | 0.006 | 0.009 | 1474 | 0.740 | 0.459 |
| Dataset 2: |  | Initial Consonant | 0.028 | 0.017 | 1406 | 1.591 | 0.112 |
| Error Type vs All Other Error Trials |  | Final Consonant | -0.019 | 0.016 | 1409 | -1.207 | 0.228 |
|  |  | **Omission** | **0.036** | **0.008** | **1572** | **4.742** | **0.000** |
|  |  | Additive | 0.001 | 0.007 | 1581 | 0.153 | 0.878 |
|  |  | Vowel | -0.005 | 0.012 | 1422 | -0.387 | 0.699 |
|  |  | Word Substitution | -0.001 | 0.004 | 1426 | -0.235 | 0.814 |

Bold text indicates factors that were significant predictors in both datasets.
